# Detecting the Non-Dipper Phenotype in Adolescents Exposed to Nighttime Screen Use—Digital Chronotoxicity as a Proposed Integrative Framework: A Narrative Review of Ambulatory Blood Pressure Monitoring, Subclinical Biomarkers, and Emerging Wearable and AI-Based Screening

**DOI:** 10.3390/diagnostics16152355

**Published:** 2026-07-27

**Authors:** Ancuta Elena Tupu, Simona Steliana Tudor, Irina Maria Tudor, Claudia Simona Stefan, Alice Elena Munteanu, Aurel Nechita

**Affiliations:** 1 Medical-Pharmaceutical Research Center, Faculty of Medicine and Pharmacy, “Dunarea de Jos” University of Galati, 800008 Galati, Romania; anca.tupu@ugal.ro (A.E.T.); claudia.stefan@ugal.ro (C.S.S.); aurel.nechita@ugal.ro (A.N.); 2 Faculty of Medicine, University of Medicine and Pharmacy “Carol Davila”, 050474 Bucharest, Romania; 3 Faculty of Medicine, “Titu Maiorescu” University, 031593 Bucharest, Romania; dralicepopescu@yahoo.com; 4 Central Military Emergency University Hospital “Dr. Carol Davila”, 010825 Bucharest, Romania

**Keywords:** ambulatory blood pressure monitoring, non-dipper phenotype, adolescents, circadian rhythm, screen time, chronodisruption, early vascular aging, pediatric hypertension

## Abstract

Arterial hypertension in adolescents is increasingly a lifestyle-driven disorder, and a growing share of cardiovascular risk in this age group is hidden from conventional office measurement. Intensive nighttime screen use, together with the chronic sleep loss that accompanies it, may disrupt circadian control of the cardiovascular system, an effect we designate, as a proposed integrative concept, digital chronotoxicity. Through melatonin suppression, sustained sympathetic and neuroendocrine activation, oxidative stress, and metabolic dysregulation, this exposure is hypothesized to attenuate the physiological nocturnal fall in blood pressure and to contribute to the non-dipper phenotype, a predictor of early vascular aging that remains invisible to office readings. This narrative review traces the mechanistic pathway from screen exposure to the loss of nocturnal dipping and reframes the problem as a diagnostic one. Twenty-four-hour ambulatory blood pressure monitoring is positioned as the reference standard for detecting the at-risk phenotype and masked hypertension; the cardiac, vascular, renal, autonomic, and neurocognitive biomarkers of early hypertension-mediated organ damage are reviewed; and emerging wearable and artificial-intelligence tools for continuous, preventive screening are examined. An integrated screening pathway is proposed, on the premise that the evaluation of a hypertensive adolescent is incomplete without a digital and sleep history alongside ambulatory monitoring. Reframed in this way, pediatric hypertension becomes a detectable chronobiological disease.

## 1. Introduction

Arterial hypertension in children and adolescents is no longer regarded as a rare disorder secondary to renal or endocrine disease, but increasingly as a silent, lifestyle-driven condition that originates in youth. Contemporary epidemiological estimates place the global prevalence of pediatric hypertension between roughly 3% and 8%, with substantially higher values among adolescents who are overweight or obese [1]. Excess adiposity remains the dominant modifiable determinant [2], yet a growing body of evidence implicates a distinct set of behavioral exposures that are now nearly universal among young people, namely prolonged screen use and chronic, partly voluntary sleep curtailment [3].

This trend was sharply accelerated by the COVID-19 pandemic. Mobility restrictions and the shift to home-based online schooling created an obesogenic, screen-saturated environment that some authors have described as covibesity, or a screen-demic [4]. Post-pandemic longitudinal data documented parallel increases in body mass index [5], while screen exposure has been associated with higher blood pressure and cardiometabolic risk in youth [6].

Adolescence is also a critical window of neurobiological development, characterized by a physiological delay in the sleep phase and by the consolidation of long-lasting sleep behaviors [7]. When this intrinsic tendency overlaps with evening use of smartphones, tablets, and laptops, the result is a progressive desynchronization between internal biological time and external behavior, a state termed chronodisruption [8]. This exposure is not biologically inert. Artificial light and emotionally engaging digital content act as false zeitgebers, or time cues, that perturb the central circadian clock [9] and, through it, the cardiovascular system, an effect that links chronodisruption to cardiovascular risk well beyond the sedentary behavior it accompanies [10].

From a hemodynamic standpoint, blood pressure follows a tightly organized circadian rhythm. Under physiological conditions, mean blood pressure falls by approximately 10% to 20% during sleep relative to daytime values, a pattern known as dipping that offloads the left ventricle and the vascular bed and provides an essential nightly recovery period [11]. Attenuation or loss of this nocturnal decline defines the non-dipper phenotype, which predicts target-organ damage and future cardiovascular events more strongly than daytime pressure alone [12]. In pediatric cohorts, the magnitude of nocturnal pressure tracks closely with early signs of end-organ injury, underscoring that the clinically decisive information resides in the sleep period rather than in the waking hours [13].

Herein lies the central diagnostic problem that motivates this review. The non-dipper phenotype, together with the related entity of masked hypertension, is by definition invisible to conventional office measurement, which samples blood pressure only during waking clinic hours; its detection requires 24 h ambulatory blood pressure monitoring (ABPM) [14]. As digital exposure reshapes the nocturnal physiology of a generation of adolescents, the question is no longer only why blood pressure is disturbed, but how the resulting circadian phenotype can be detected early, before it becomes clinically overt.

**Figure 1:**
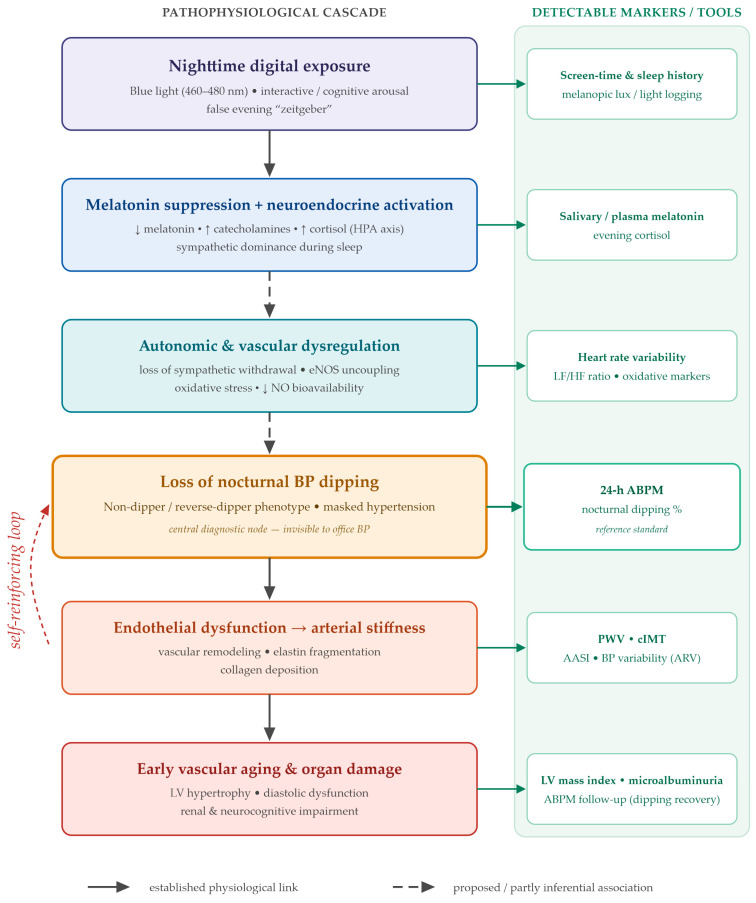
The digital chronotoxicity cascade and its detectable markers.

This narrative and integrative review addresses that question through a diagnostic lens. We propose the unifying concept of digital chronotoxicity, defined as the cumulative cardiovascular cost of the intensity, timing, and content of nighttime screen exposure, and we trace the proposed pathway through which it may suppress melatonin, sustain sympathetic and neuroendocrine activation, and attenuate physiological dipping, potentially culminating in early vascular aging (Figure 1). We emphasize at the outset that digital chronotoxicity is advanced as an integrative, hypothesis-generating construct rather than an established clinical diagnosis. Against this mechanistic background, the review focuses on detection: the role of ABPM as the reference standard, the subclinical biomarkers that capture early hypertension-mediated organ damage, and the emerging wearable and artificial-intelligence tools that may enable preventive, real-time screening in this uniquely vulnerable population.

## 2. Materials and Methods

This article was written as a narrative and integrative review, a format chosen because the subject draws on heterogeneous bodies of evidence, including experimental and molecular work, observational and clinical pediatric studies, ambulatory blood pressure cohorts, clinical guidelines, and earlier reviews, that are best brought together through conceptual synthesis rather than quantitative pooling. The aim was to integrate these strands into a single, diagnostically oriented account rather than to estimate a pooled effect, and the synthesis was guided by recognized criteria for the quality of narrative review articles [15].

The literature was identified through structured searches of PubMed, Scopus, Web of Science, and Google Scholar covering January 2009 to June 2026 (final search performed on 5 June 2026). The lower bound corresponds to the year in which chronodisruption was first formally defined; a small number of seminal earlier sources (for example, on blood pressure tracking and on sleep and metabolism) were retained where foundational. Search strategies were adapted to each database and combined controlled vocabulary with free-text terms using Boolean operators. In PubMed, the core strategy was: (“pediatric hypertension” OR “adolescent hypertension” OR “non-dipper” OR “nocturnal blood pressure” OR “ambulatory blood pressure monitoring”) AND (“circadian rhythm” OR chronodisruption OR “sleep quality” OR “sleep deprivation” OR “screen time” OR “screen exposure” OR “blue light” OR melatonin OR “sympathetic nervous system”). Studies were eligible if they addressed at least one review domain, the circadian physiology of blood pressure, the pathophysiology of digital chronotoxicity, the diagnostic evaluation of the non-dipper phenotype, or emerging screening technologies, and were clinical, observational, experimental, meta-analytical, or guideline sources. Records were excluded if full text was unavailable, if they were unrelated to the digital-exposure–circadian–cardiovascular axis, or if they were duplicates. Two authors screened titles and abstracts for relevance and then assessed full texts for eligibility, with duplicates removed before full-text review and disagreements resolved by discussion. A final set of 90 sources was retained and organized thematically into the domains that structure this review. Throughout this review, the term “adolescents” refers to individuals aged approximately 10–19 years; where the cited evidence derives from broader pediatric samples, from young adults, or from experimental adult paradigms, this is indicated, and such data are treated as supportive rather than as direct evidence in adolescents. Sources were selected on the basis of their relevance to the central theme, namely the relationship between digital exposure, circadian disruption, and the cardiovascular phenotype of the adolescent, and were then organized thematically into the domains that structure this review: the circadian physiology of blood pressure regulation, the pathophysiology of digital chronotoxicity, the diagnostic evaluation of the non-dipper phenotype, and emerging technologies, screening, and management. In keeping with the narrative design, no formal risk-of-bias assessment or quantitative synthesis was undertaken; greater interpretive weight was given to primary controlled, longitudinal, and ambulatory-monitoring-based pediatric studies than to narrative reviews, and the limitations of the evidence base are addressed throughout.

## 3. Pathophysiology of Digital Chronotoxicity and Circadian Blood Pressure Disruption

**Figure 2:**
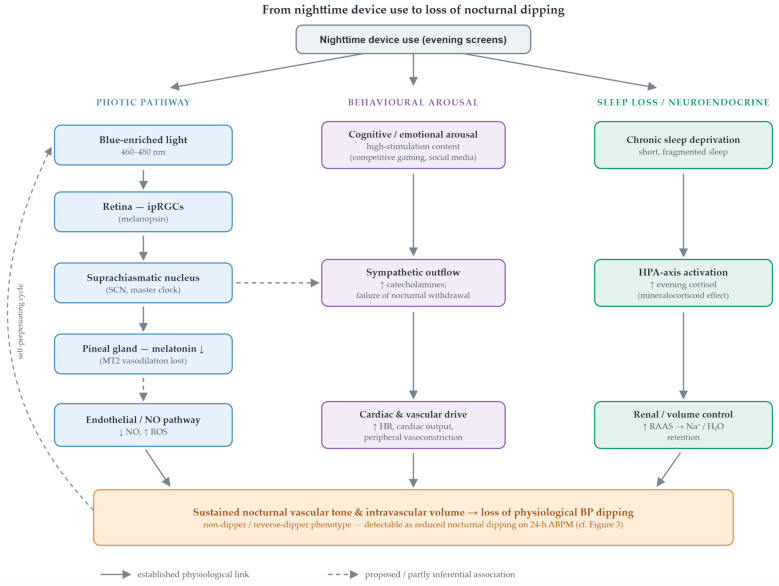
Convergent mechanistic axes (photic circadian signaling and behaviorally induced arousal) linking nighttime device use to loss of nocturnal dipping. Solid arrows denote established physiological links; dashed arrows denote proposed or partly inferential associations.

The association between digital exposure and an altered blood pressure profile is best understood as the plausible product of several converging, mutually reinforcing mechanisms. Understanding them must begin with the physiology that generates the nocturnal blood pressure fall, because digital chronotoxicity is, in mechanistic terms, the progressive dismantling of that physiology (Figure 2).

### 3.1. Physiology of Nocturnal Blood Pressure Regulation

The circadian variation in blood pressure is not a passive consequence of rest but an actively orchestrated process governed by the autonomic nervous system and by hormonal axes that are themselves under circadian control [11]. Its anatomical pacemaker is the suprachiasmatic nucleus (SCN) of the anterior hypothalamus, which receives photic information directly from the retina through the retinohypothalamic tract and synchronizes the peripheral molecular clocks operating in almost every cell, including cardiomyocytes and vascular endothelial cells [16]. At the molecular level, this timing is generated by a transcriptional and translational feedback loop in which the CLOCK and BMAL1 proteins activate transcription of the period (PER) and cryptochrome (CRY) genes; the accumulating PER and CRY proteins then inhibit the CLOCK-BMAL1 complex and, through their gradual overnight degradation, close a cycle of approximately 24 h [16]. Because this oscillator controls the transcription of genes governing catecholamine synthesis, angiotensin-converting enzyme activity, and renal sodium handling, its disruption dysregulates vascular tone and hydrosaline balance independently of the sleep or wake state, defining the temporal windows of cardiovascular vulnerability [11].

During the transition from wakefulness to non-rapid-eye-movement sleep, the sympathovagal balance shifts decisively. A resetting of the baroreflex accompanies a phenomenon of sympathetic withdrawal, with reduced adrenergic discharge to the heart and vessels and a corresponding rise in parasympathetic tone [17]. The hemodynamic consequences of this autonomic shift are precisely those that produce dipping: cardiac output falls through reductions in heart rate and stroke volume, and total peripheral resistance falls through systemic vasodilation [17].

Melatonin is a frequently overlooked but central component of this nocturnal hemodynamic program. Secreted by the pineal gland under SCN control and only in darkness, it reaches peak concentrations between approximately 02:00 and 04:00 [18]. Beyond its hypnotic role, melatonin acts directly on the vasculature through the G-protein-coupled receptors MT1 and MT2 expressed on vascular endothelium and smooth muscle; activation of MT2 receptors in the peripheral vascular bed promotes vasodilation [19]. Mechanistically, melatonin raises cytosolic calcium in endothelial cells and activates endothelial nitric oxide synthase, increasing the production of nitric oxide, the principal endogenous vasodilator [18]. In experimental and pharmacological models, melatonin also scavenges reactive oxygen species; by limiting superoxide-driven inactivation of nitric oxide, this activity has been proposed to help preserve nitric oxide bioavailability. This mechanism is, however, largely derived from preclinical and adult data rather than from adolescent cohorts, and is presented here as a plausible contributory pathway rather than an established nocturnal effect [20].

Volume control during sleep is governed in parallel by the renin–angiotensin–aldosterone system, whose activity follows a circadian rhythm opposite to that of melatonin and is suppressed during deep sleep. The resulting decline in plasma renin activity lowers angiotensin II and aldosterone, and the fall in nocturnal aldosterone allows the kidney to shift from daytime sodium retention toward pressure natriuresis, eliminating the sodium and water accumulated during the day and reducing intravascular volume [21,22]. In adolescents, this equilibrium is fragile. Pubertal increases in growth hormone and sex steroids interact with autonomic control and modulate the 24 h pressure profile [23], so that any nocturnal arousal, whether from a notification, ambient light, or psychosocial stress, can instantly reactivate the sympathetic axis, stimulate renin release, and block natriuresis, thereby sustaining elevated nighttime pressure [7,17].

### 3.2. Blue Light and Melatonin Suppression

Contemporary electronic displays emit short-wavelength light enriched in the blue portion of the spectrum, around 460 to 480 nm, the very wavelengths that most strongly excite melanopsin, the photopigment of the intrinsically photosensitive retinal ganglion cells (ipRGCs). Unlike the rods and cones that subserve image formation, ipRGCs transmit luminance information directly to the SCN through the retinohypothalamic tract, and their activation prompts the SCN to inhibit the pineal gland and suppress melatonin secretion even at relatively low light intensities [24]. Evening exposure to such light therefore acts as a potent nocturnal signal of daytime.

The functional consequence is a measurable suppression and phase delay of melatonin. Controlled studies have shown that the use of light-emitting screens in the hours before sleep reduces and delays the evening melatonin rise [25,26], and this effect appears more pronounced in adolescents than in adults. Two converging mechanisms explain this pubertal vulnerability. First, the circadian system is intrinsically more sensitive to evening light during early puberty and mid-puberty, so that a given light dose is associated with greater melatonin suppression in younger individuals [27]. Second, the crystalline lens of the young eye is more transparent to short-wavelength light, transmitting more blue light to the retina than the progressively yellowing adult lens [28]. The result is a developmental window in which nighttime device use exerts an outsized chronodisruptive effect, sustaining a self-perpetuating cycle of light exposure, melatonin loss, and delayed sleep that is characteristic of adolescence [29].

The hemodynamic implications follow directly from the physiology described above. Suppressing melatonin removes the MT2-mediated vasodilation that normally lowers nocturnal vascular tone [18] and may withdraw the antioxidant protection thought to help preserve nitric oxide bioavailability through the night [20]. The vascular bed is consequently held at a higher tone during the very hours when it should be relaxing, which opposes the nocturnal blood pressure fall and biases the 24 h profile away from the dipper pattern [11].

### 3.3. Cognitive Hyperarousal and Sympathetic Activation

Light is only one of the two pathways through which evening device use disturbs nocturnal hemodynamics; the second is the content itself. Competitive video games and social media platforms are engineered around reward algorithms that elicit phasic dopamine release in the nucleus accumbens, the core of the brain’s reward circuitry [30]. This interactive, reward-driven engagement is considerably more arousing, and more disruptive to subsequent sleep, than passive screen exposure such as watching a film [31,32,33], and the cognitive and emotional activation it leads to is fundamentally incompatible with the parasympathetic quiescence that sleep onset requires [34].

The consequences extend beyond delayed sleep. Rather than reflecting a simple biosynthetic conversion of reward-related dopamine into circulating norepinephrine, the cardiovascular effect is better understood as central arousal driving autonomic imbalance. Engaging, reward-driven content sustains cognitive and emotional activation that delays the normal evening shift toward parasympathetic dominance, so that the adolescent remains in a state of heightened sympathetic tone at the very time when adrenergic activity should be declining. Heart rate and vascular tone are thereby held closer to daytime levels through the early night. Two partly independent inputs therefore converge on nocturnal sympathetic tone: the photic, melatonin-mediated circadian pathway, and behaviorally induced arousal from high-stimulation content such as competitive gaming immediately before sleep, which raises sympathetic activity independently of light. Persistent sympathetic activation of this kind is not benign in the young: it has been associated with subclinical target-organ damage in young adults, linking the behavioral exposure to early vascular injury [35].

This autonomic imbalance is measurable. Heart rate variability, a recognized marker of autonomic balance and stress [36], is well suited to capture this shift; evening screen use has been associated with a relative increase in the low-frequency to high-frequency ratio and reduced vagal tone. This ratio is often read as a shift toward sympathetic predominance and reduced vagal tone, but it is not a direct or specific index of sympathovagal balance: it is influenced by respiration rate, posture, sleep stage, recording duration, and the signal-processing method, and should therefore be treated as a non-specific autonomic marker rather than a quantitative measure of sympathetic activity. A blunted nocturnal recovery of vagal activity keeps heart rate and blood pressure elevated during sleep, providing a quantifiable link between evening cognitive hyperarousal and the loss of the nocturnal blood pressure fall. Heart rate variability thus emerges as a mechanistic and exploratory candidate marker, not a specific diagnostic test for the non-dipper phenotype, a point developed further in the discussion of wearable monitoring.

### 3.4. Sleep Deprivation and HPA Axis Dysregulation

The light and arousal mechanisms described above share a common behavioral endpoint: a chronic shortening of total sleep duration. Across school-aged children and adolescents, greater screen use is consistently associated with later bedtimes and reduced sleep [37,38,39,40], an effect that is particularly marked for portable, interactive devices used in bed [41]. Insufficient sleep is not merely a deficit of rest; it is a recurring physiological stressor.

Chronic sleep curtailment activates the hypothalamic–pituitary–adrenal (HPA) axis, the body’s principal neuroendocrine stress system [42]. The characteristic result is an elevation of serum cortisol during the evening and night, precisely the window in which cortisol should reach its circadian nadir. Because cortisol exerts mineralocorticoid activity at the renal tubule, this nocturnal hypercortisolemia promotes sodium and water retention, expanding intravascular volume and raising arterial pressure during sleep [42]. Sleep loss simultaneously shifts autonomic balance toward sympathetic predominance, so the neuroendocrine and autonomic limbs reinforce one another, and both oppose the volume contraction and vasodilation that normally produce nocturnal dipping [43].

Disturbed and insufficient sleep has also been associated with a low-grade systemic inflammatory state, including elevations in high-sensitivity C-reactive protein and interleukin-6. The magnitude and direction of this association vary with the nature of the exposure—acute total sleep deprivation, chronic partial sleep restriction, and circadian misalignment are not equivalent—and are further confounded by adiposity, which is both a cause of disturbed sleep and an independent driver of inflammation, so that the contribution of sleep loss itself is partial and difficult to isolate [44]. Where present, these mediators plausibly link disturbed sleep to vascular injury, because they drive the generation of reactive oxygen species that, as described in the following section, can uncouple endothelial nitric oxide synthase and accelerate vascular damage.

### 3.5. Oxidative Stress and Endothelial Dysfunction

The pro-inflammatory milieu generated by chronic sleep loss converges on the vascular endothelium through oxidative stress. Inflammatory mediators stimulate the production of reactive oxygen species, of which the superoxide anion is the most consequential for vascular tone [44]. Superoxide reacts rapidly with nitric oxide to form peroxynitrite, a highly reactive and cytotoxic molecule, and in doing so initiates a self-amplifying process known as eNOS uncoupling, in which endothelial nitric oxide synthase shifts from generating vasoprotective nitric oxide to generating still more superoxide [44].

The hemodynamic cost of this shift is twofold. First, the bioavailability of nitric oxide falls, impairing endothelium-dependent vasodilation at the very time of night when vasodilation should predominate; the simultaneous loss of melatonin, whose antioxidant action is thought to help shield nitric oxide from degradation, may remove a compensatory defense and deepen the deficit [20]. Second, sustained oxidative and inflammatory injury is not confined to vascular tone but drives structural remodeling of the arterial wall, an effect consistent with the broader association between circadian disruption and adverse cardiometabolic and vascular outcomes [45].

Over time, this remodeling manifests as increased arterial stiffness, a hallmark of vascular aging and a recognized correlate of hypertensive phenotypes [46]. The two consequences are mutually reinforcing: a stiffer, less compliant vasculature with reduced nitric oxide reserve cannot relax adequately during sleep, which both anchors the non-dipper profile and accelerates the very stiffening that produced it. In this way, the molecular events set in motion by nighttime digital exposure translate into a measurable structural trajectory toward early vascular aging, the biomarkers of which are the subject of the diagnostic evaluation that follows.

### 3.6. Metabolic Dysregulation

A frequently underappreciated arm of digital chronotoxicity is its effect on metabolism, where chronodisruption, glucose handling, and blood pressure homeostasis are linked bidirectionally. Curtailed and fragmented sleep deregulates peripheral metabolism within only a few nights, reducing insulin sensitivity and creating a pro-hypertensive internal environment [42,47]. In adolescents and youth, shorter and poorer sleep is associated with an adverse cardiometabolic profile [48], and disturbed sleep is a plausible link between screen exposure and emerging cardiometabolic risk [49], representing the pivotal link between behavior and metabolic injury [50].

The decline in insulin sensitivity provokes a compensatory hyperinsulinemia that is itself a pressor. Insulin stimulates sympathetic nervous activity and enhances tubular sodium reabsorption, so that the adolescent with sleep-induced insulin resistance enters a state of chronic sodium and water retention in which an expanded intravascular volume becomes a primary driver of salt-sensitive hypertension [21,42]. This volume-dependent mechanism aligns with, and compounds, the autonomic and neuroendocrine pathways described earlier.

Sleep restriction also disturbs the hormonal control of appetite, lowering the satiety hormone leptin and raising the hunger hormone ghrelin, and it engages reward-related pathways that promote a hedonic drive toward energy-dense foods rich in refined carbohydrates and salt during the nocturnal waking period [42]. Evening salt intake is particularly detrimental to the blood pressure profile, because the kidney must maintain a high perfusion pressure overnight to excrete the ingested sodium load, directly opposing the physiological nocturnal fall [21]. Taken together, the metabolic arm converges with the melatonin, autonomic, neuroendocrine, and oxidative pathways on a single hemodynamic outcome, the loss of nocturnal dipping, while contributing its own detectable signature in the form of insulin resistance and an adverse cardiometabolic profile. These convergent mechanisms and the markers that detect them are summarized in Table 1.

## 4. Diagnostic Evaluation: ABPM and Biomarkers of Early Organ Damage

### 4.1. Circadian Blood Pressure Phenotypes: Definitions and Diagnostic Criteria

The recognition that nighttime blood pressure carries greater prognostic weight than daytime values for cardiovascular morbidity and mortality has made the circadian blood pressure phenotype, rather than the office reading alone, the clinically decisive variable [12]. Contemporary guidelines define the phenotype by the magnitude of the nocturnal decline in mean blood pressure relative to daytime values. A physiological fall of at least 10% defines the dipper pattern, whereas a fall of less than 10% defines the non-dipper phenotype [52]. Two further categories refine the spectrum: the extreme dipper, with an exaggerated nocturnal fall exceeding 20%, and the reverse dipper or riser, in which mean nighttime pressure exceeds daytime pressure. In pediatric practice, the assessment and classification of these patterns are codified by dedicated ambulatory blood pressure monitoring standards for children and adolescents [53]. These circadian dipping phenotypes must be distinguished from two related but non-identical entities. Nocturnal hypertension denotes an absolute asleep blood pressure above the age- and height-specific threshold and can coexist with, but is not equivalent to, a blunted dip: reduced dipping may occur while absolute nighttime pressure remains within the normal range, and, conversely, nocturnal hypertension can be present in a preserved-dipper profile. Masked hypertension is defined by normal office readings together with elevated out-of-office (ambulatory or home) pressure, and is a distinct diagnostic category that may or may not be accompanied by non-dipping. Throughout this review these terms are used in their specific senses and are not treated as interchangeable.

The clinical importance of the non-dipper phenotype follows from its pathophysiology. The absence of the nocturnal pressure fall maintains a continuous hemodynamic load on the arterial wall and the myocardium and deprives the cardiovascular system of the baroreflex recovery period that sleep should provide. This sustained nocturnal burden is not a benign laboratory finding: blood pressure tracks from childhood into adulthood, and adolescents with a non-dipping profile carry a higher probability of developing established hypertension as adults [54,55].

**Figure 3:**
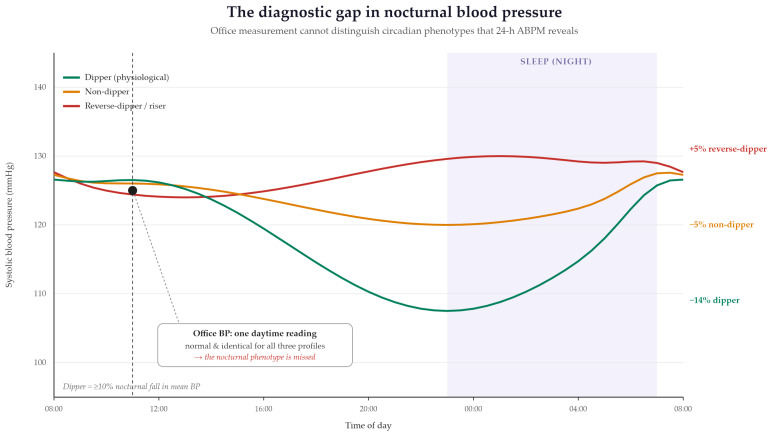
The diagnostic gap between office blood pressure and 24 h ABPM. This is an illustrative schematic constructed for explanatory purposes and is not derived from patient data.

The reverse-dipper, or riser, phenotype represents the most adverse end of the spectrum, because nocturnal pressure that exceeds daytime pressure confers the highest cardiovascular risk of all circadian patterns [56]. This is of particular relevance to the present review, and it is plausible, based on the mechanisms described above, that adolescents with disordered sleep and heavy nighttime screen use are more likely to show the riser pattern, although this has not been established by direct pediatric data. The circadian phenotypes are illustrated in Figure 3 and, with their defining criteria and prognostic significance, are summarized in Table 2.

### 4.2. Ambulatory Blood Pressure Monitoring as the Diagnostic Reference Standard

Conventional office measurement is structurally incapable of detecting the abnormalities that matter most in this setting. Because it samples blood pressure only during waking clinic hours, it cannot quantify the nocturnal period, cannot reveal an attenuated or reversed dip, and cannot identify masked hypertension, in which office readings are normal while out-of-office pressure is elevated. For these reasons, current pediatric standards position 24 h ambulatory blood pressure monitoring (ABPM) as the reference method for diagnosing and classifying the circadian phenotype in children and adolescents [53], a position reinforced by European pediatric hypertension guidance [14].

ABPM records oscillometric readings at fixed intervals across a full 24 h cycle, partitioning them into awake and asleep periods, ideally defined by a concurrent sleep diary, and computing the nocturnal dipping ratio as the percentage fall in mean pressure from the awake to the asleep period. The accuracy of this assessment depends on the use of devices validated against recognized international standards, since unvalidated instruments can misclassify the phenotype [57]. Beyond mean values, ABPM yields derived indices, including pulse pressure, the ambulatory arterial stiffness index, and the average real variability of blood pressure, that add prognostic information and are increasingly characterized in pediatric cohorts [58]. The nocturnal dipping ratio is computed as the percentage fall in mean pressure from the awake to the asleep period—dipping (%) = (mean awake BP − mean asleep BP)/mean awake BP × 100—and is derived separately for systolic and diastolic pressure, since the two do not always fall in parallel and may yield discordant phenotypes. A valid pediatric recording conventionally requires an adequate proportion of successful readings (commonly ≥70–80%) obtained at fixed intervals (typically every 15–20 min when awake and every 20–30 min when asleep), with an appropriately sized cuff and a device validated for pediatric use against a recognized protocol [53,57]. The awake and asleep periods are ideally defined from a concurrent sleep diary or actigraphy rather than from fixed clock times. A caveat specific to this population deserves emphasis. In adolescents who use digital media late into the night, the clock-defined nighttime interval may not coincide with the period of actual sleep. A reduced or absent dip computed against a fixed nocturnal window can therefore partly reflect ongoing wakefulness, gaming, or emotional arousal and a delayed sleep onset, rather than a true failure of the sleeping cardiovascular system to relax. Defining the asleep period from a concurrent sleep diary, actigraphy, or a device-derived sleep window, rather than from fixed clock times, is consequently not a technical refinement but a prerequisite for interpreting the dipping ratio correctly in this group [59,60].

The diagnostic yield of ABPM is clinically consequential. In children, ambulatory measures correlate with end-organ damage more closely than office values, an association that is especially evident in higher-risk groups such as those with obesity [13]. ABPM also reframes the goal of treatment: therapeutic success is most meaningfully defined by the restoration of physiological nocturnal dipping rather than by the normalization of daytime readings alone, making the asleep blood pressure both the principal diagnostic target and the principal therapeutic target [12,61].

These strengths are balanced by practical limitations. ABPM can be poorly tolerated by younger patients, nocturnal cuff inflations may themselves disturb sleep, and the reproducibility of the dipping classification across nights is imperfect, so a single study can misclassify a borderline phenotype. Home blood pressure monitoring offers a more accessible, repeatable adjunct, although it cannot capture the asleep period that defines dipping. The comparative capabilities and limitations of office measurement, home monitoring, ABPM, and emerging wearable approaches are summarized in Table 3.

### 4.3. Cardiac Biomarkers of Early Organ Damage

The principal consequence of an unremitting nocturnal pressure load is hypertension-mediated organ damage.

It should be emphasized that melatonin, cortisol, inflammatory indices (hs-CRP, IL-6), insulin-resistance measures, heart rate variability, and neurocognitive performance are mechanistic or supportive markers rather than specific diagnostic biomarkers of the non-dipper phenotype, which is defined solely by ambulatory blood pressure monitoring. The heart is among the earliest organs to register this burden. The left ventricular mass index correlates more closely with nocturnal than with daytime pressure, and a higher nocturnal pressure load, of which an attenuated dip is one component, has been associated with left ventricular hypertrophy in hypertensive children and adolescents; the extent to which isolated non-dipping, in the absence of elevated nocturnal pressure, independently drives hypertrophy is less well established [62].

Left ventricular hypertrophy is not merely a structural curiosity in the young; it is an established independent predictor of major cardiovascular events, including myocardial infarction and stroke, in early adulthood, which gives its detection in adolescence genuine prognostic weight [62]. Importantly, structural hypertrophy is preceded by functional change. Tissue Doppler imaging reveals early diastolic dysfunction in non-dipper adolescents before overt hypertrophy is apparent, so sensitive echocardiographic assessment can identify cardiac involvement at a stage when it may still be reversible with circadian and lifestyle correction [63]. Echocardiographic indices of left ventricular mass and diastolic function therefore represent accessible, clinically validated biomarkers that translate the abstract dipping ratio into measurable cardiac risk.

### 4.4. Vascular Biomarkers of Early Organ Damage

Sustained exposure to elevated nocturnal pressure also remodels the vascular wall, driving elastin fragmentation and collagen deposition that stiffen the large arteries; in the pediatric setting, these changes are captured within the broader framework of hypertension-mediated organ damage [63]. The reference measure of this process is pulse wave velocity, the speed at which the pressure wave propagates along the aorta, which rises as the vessel stiffens. Higher pulse wave velocity has been reported in youth with elevated blood pressure and adverse hemodynamic profiles; where a non-dipping pattern has specifically been examined, it has likewise been associated with increased arterial stiffness, although much of the pediatric evidence reflects overall hypertension severity or nocturnal pressure load rather than isolated dipping status [64]. Arterial stiffening is self-perpetuating. A stiffer aorta loses its capacity to buffer the pulse wave, which raises central pressure, and the higher pressure in turn accelerates further stiffening, establishing a vicious cycle that is difficult to reverse once it takes hold during adolescence [46]. Carotid intima–media thickness provides a complementary structural marker of this early arteriopathy and can be assessed noninvasively in young patients [63].

Because dedicated imaging is not always available, ABPM itself supplies surrogate vascular indices. The ambulatory arterial stiffness index and the average real variability in blood pressure, both derived directly from the 24 h recording, track arterial stiffness and pressure lability and are increasingly reported in pediatric cohorts [58]. Elevated blood pressure variability has been linked to a low-grade inflammatory state in children with primary hypertension, reinforcing the mechanistic continuity between the oxidative and inflammatory pathways of digital chronotoxicity and their measurable vascular consequences [65]. Together, pulse wave velocity, carotid intima–media thickness, and the ambulatory stiffness and variability indices constitute a layered panel for detecting vascular aging before it becomes clinically irreversible.

### 4.5. Renal Biomarkers of Early Organ Damage

An elevated nocturnal pressure load may affect the kidney as it does the heart and vasculature, and this renal involvement is most relevant where a non-dipping profile coincides with raised nighttime pressure. The pathogenic mechanism is nocturnal glomerular hyperfiltration: deprived of the physiological pressure fall, the kidney is forced to filter at an elevated intraglomerular pressure during a period when it should be in relative functional rest. This sustained hyperfiltration injures the podocytes and manifests as microalbuminuria, which serves as an early and accessible marker of renal involvement and, because the glomerular endothelium reflects the systemic vascular bed, as an indicator of generalized endothelial dysfunction [63,66].

Microalbuminuria therefore links the renal and vascular axes of organ damage into a single, measurable signal. In pediatric cohorts, elevated albuminuria has been related chiefly to the overall nocturnal pressure load and hypertension severity, and its specific association with isolated non-dipping is less firmly established; where present in an adolescent with a non-dipping profile, it nonetheless suggests that the nocturnal pressure load is already exacting a structural cost. The same circadian dysregulation of the renin–angiotensin–aldosterone system that sustains nocturnal volume expansion also contributes to this renal burden, so that plasma renin and aldosterone profiles together with ambulatory variability indices add complementary information about target-organ involvement in pediatric hypertension [22,58]. Spot urinary albumin measurement is inexpensive and noninvasive, which makes it well suited to the screening orientation of this review.

### 4.6. Autonomic and Neurocognitive Markers

Two further domains extend the diagnostic panel beyond the cardiovascular and renal systems. The first is autonomic. As discussed in relation to evening hyperarousal, heart rate variability provides a noninvasive, if indirect, window into autonomic regulation; an increased low-frequency to high-frequency ratio is commonly associated with reduced vagal tone, though, as noted above, it is a non-specific marker whose interpretation depends on recording conditions and processing [36]. Because heart rate variability can be recorded continuously and unobtrusively, it is among the most promising candidate markers for the screening applications considered later in this review, bridging mechanism and measurement.

The second domain is neurocognitive. Primary hypertension in children and adolescents has been associated with lower performance on tests of attention and executive function, indicating that the consequences of an elevated pressure burden reach the developing brain [67]; this evidence concerns hypertension broadly rather than dipping status specifically. The proposed substrate is injury to the cerebral white matter and microcirculation, although the pathophysiology is not yet fully elucidated; circadian disruption itself has independently been implicated in brain function and may compound the vascular contribution [68]. While neurocognitive assessment is not a routine diagnostic test for the non-dipper phenotype, its inclusion underscores that the stakes of undetected nocturnal hypertension in adolescence extend to cognitive development, strengthening the argument for early detection. The complete panel of biomarkers spanning the cardiac, vascular, renal, autonomic, and neurocognitive domains is consolidated in Table 4.

## 5. Emerging Technologies, Integrated Screening, and Clinical Management

### 5.1. Wearable Devices for Continuous Monitoring

The diagnostic methods described so far provide accurate but intermittent snapshots; the next advance lies in continuous, real-world monitoring. Consumer and research-grade wearable devices can track heart rate, heart rate variability, skin conductance, peripheral temperature, and actigraphy-derived sleep across many consecutive days, and validation studies confirm that the better instruments capture sleep and cardiac parameters with acceptable agreement against reference methods [69]. This capability is directly relevant to digital chronotoxicity, because the autonomic disturbance that precedes the loss of nocturnal dipping is expressed precisely in the heart rate variability metrics that wearables measure unobtrusively in the adolescent’s own environment [36].

The appeal of these devices in a young population is threefold. They are worn willingly and continuously, generating longitudinal data that a single ambulatory study cannot provide; they capture the night-to-night variability that is itself informative; and they situate measurement in the everyday context in which digital exposure actually occurs. Their principal limitation concerns blood pressure itself: cuffless and photoplethysmography-based estimates are not yet validated to the standard required of ambulatory monitors, so wearables presently complement rather than replace ABPM for phenotype classification [57]. Several biomedical-engineering constraints further bound what wearables can deliver. Cuffless, photoplethysmography-based blood pressure estimates require individual calibration and are subject to calibration drift over time, so they cannot substitute for a validated cuff measurement, and no consumer device is yet validated for non-dipping classification against ABPM in a pediatric population. Photoplethysmographic signals are additionally degraded by motion artifacts, variable contact pressure and sensor placement, posture and sleep position, skin temperature, skin pigmentation, and missing data during the night; pulse-rate variability derived from photoplethysmography is not interchangeable with electrocardiographically derived heart rate variability. Wearable heart rate variability, actigraphy, and sleep metrics are therefore best positioned as tools for longitudinal risk screening and context, not as diagnostic substitutes for ambulatory blood pressure measurement, until pediatric validation and calibration standards are met. The value of the continuous data streams they generate is realized fully only when those data are analyzed by algorithms capable of detecting patterns invisible to episodic assessment, which is the subject of the following section.

### 5.2. Artificial Intelligence, Predictive Models, and Digital Biofeedback

Continuous monitoring becomes clinically powerful only when paired with analytics able to interpret it. Machine-learning algorithms can learn complex, individualized patterns from the dense data generated by wearables and smartphones and relate them to ambulatory blood pressure profiles, raising the prospect of early-warning systems that flag emerging risk before it is clinically evident [70]. In the specific context of adolescent hypertension, such models could correlate digital inputs, including the duration of gaming sessions, screen brightness, and the time of the last evening interaction, with physiological outputs such as heart rate variability and sleep quality [36], and could alert a young user that the current pattern of evening screen use predisposes to a non-dipping night. Such a system does not yet exist for adolescent non-dipping; if developed and prospectively validated, this form of digital biofeedback could shift management from a reactive posture toward a preventive one grounded in real-time behavior modification [70].

These possibilities represent a research direction rather than an established capability. The available work in adjacent areas does not validate AI prediction of ABPM-confirmed non-dipping in adolescents; it demonstrates only the broader feasibility of machine learning in young cardiovascular populations, for example in enhancing interpretation of the resting electrocardiogram in young athletes [71], in integrating multimodal anthropometric, physiological, and biological data in adolescent cohorts [72], and in linking quality-of-life and clinical–demographic factors with cardiac outcomes [73]. Translating these methods into a tool for detecting the non-dipper phenotype would require, at a minimum, adequately sized ABPM-labeled pediatric datasets, external and prospective validation, formal calibration assessment, evaluation of algorithmic fairness across relevant subgroups, and explicit reporting of false-positive and false-negative rates before any clinical deployment. With these safeguards, continuous sensing and algorithmic interpretation may eventually support precision chronocardiology for individualized, anticipatory detection of the at-risk phenotype.

### 5.3. Genetic Susceptibility and Chronotype Screening

Not every adolescent exposed to intensive nighttime screen use develops a non-dipping profile, which points to substantial individual variability in susceptibility and creates an opening for targeted screening. Part of this variability is genetic. The molecular clock that governs cardiovascular timing is built from a small set of core genes, including *CLOCK*, *BMAL1*, and *PER2* [16], and common variation in the genetic architecture of sleep and circadian traits has been mapped in large population studies [74]. Profiling such variation could in principle identify adolescents whose circadian systems are intrinsically more vulnerable to chronodisruption, and therefore most likely to translate digital exposure into cardiovascular harm.

A more immediately accessible axis of susceptibility is chronotype. Adolescents with an evening chronotype, who are naturally inclined toward later sleep and are correspondingly more exposed to evening light, appear more vulnerable to the metabolic and cardiovascular consequences of circadian misalignment than their morning-type peers [75]. The behavioral expression of this misalignment, the discrepancy between sleep timing on school days and free days known as social jetlag, has been associated with adverse cardiometabolic traits as early as in young adolescence [76]. Because chronotype and social jetlag can be assessed with brief validated questionnaires rather than laboratory testing, they offer a practical, low-cost means of stratifying risk at the population level [9]. The combination of inexpensive chronotype screening with selective genetic profiling embodies the precision-medicine logic that should increasingly guide which adolescents receive intensive lifestyle intervention and ambulatory monitoring.

### 5.4. Quantifying Exposure: Melanopic Light and Circadian-Safe Screens

If digital chronotoxicity is to be detected and managed, the exposure itself must be measured meaningfully, and conventional photometry is poorly suited to the task. Standard illuminance, expressed in lux, weights light according to its visual brightness rather than its circadian effect. The biologically relevant metric is melanopic light, which weights radiation by its capacity to stimulate the melanopsin-containing ipRGCs that drive the circadian system, and consensus frameworks now express recommended evening and nighttime light limits in these terms [77]. Adopting melanopic quantification would allow exposure to be characterized in the units that actually predict melatonin suppression.

This reframing exposes the limitations of current mitigation strategies. Software features that shift the display toward warmer color temperatures, such as the widely used night modes, reduce blue emission but do not reliably prevent melatonin suppression when overall screen brightness remains high [78], because the magnitude of suppression depends jointly on intensity and spectral content rather than on color temperature alone [79]. Dedicated blue-light-blocking interventions, including filtering lenses worn in the evening, can partially preserve melatonin and improve sleep, although they address only the photic and not the cognitive-arousal component of digital chronotoxicity [80]. The wavelengths and intensities at issue are the same short-wavelength signals that suppress melatonin through the retinohypothalamic pathway [24,25].

The practical implication is the need to define a melanopic threshold below which the adolescent circadian system remains undisturbed. Establishing such a threshold would make it possible to certify circadian-safe displays and to specify evidence-based limits for evening device use, converting a vague behavioral recommendation into a measurable exposure target.

### 5.5. An Integrated Screening Pathway and Public Health Implications

**Figure 4:**
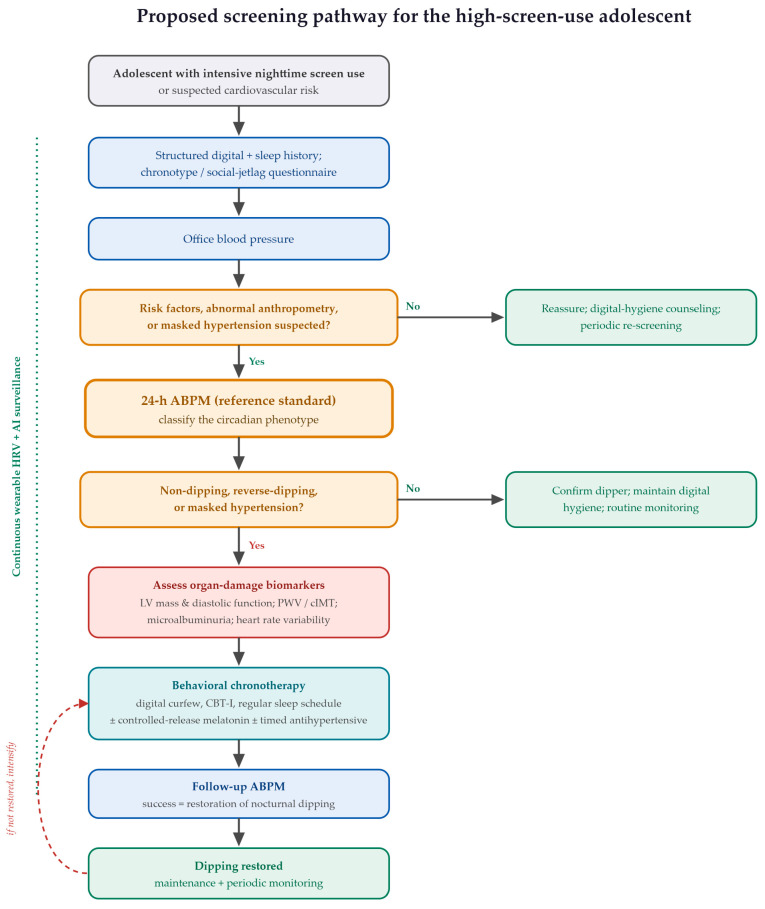
Proposed screening pathway for the high-screen-use adolescent. The pathway is a conceptual framework proposed by the authors; heavy nighttime screen exposure alone is not, at present, an established indication for ambulatory blood pressure monitoring or organ-damage assessment, which remain guided by recognized clinical criteria.

The elements assembled in this review can be organized into a practical, stepwise screening pathway for the adolescent with heavy nighttime digital exposure (Figure 4). The entry point is a structured history that quantifies screen use and sleep and incorporates a brief chronotype or social-jetlag questionnaire, identifying the behavioral and circadian risk profile at minimal cost [7,76]. Office blood pressure follows, but with explicit recognition that a normal reading does not exclude the phenotype of concern. Escalation to 24 h ABPM should follow recognized clinical indications, abnormal or high-normal office blood pressure, suspected masked hypertension, obesity, sleep-disordered breathing, chronic kidney disease, or other high-risk conditions, for which ABPM is the definitive test of the circadian phenotype [14,53]. The digital and sleep history refines risk stratification and may heighten suspicion, but heavy screen exposure alone does not, on current evidence, constitute an independent indication for ambulatory monitoring. A confirmed non-dipping pattern, particularly when accompanied by nocturnal or ambulatory hypertension or another high-risk feature, then prompts targeted assessment of hypertension-mediated organ damage [63].

Meanwhile, continuous wearable monitoring of heart rate variability, and prospectively algorithmic risk flagging, can provide longitudinal surveillance between formal studies [69,70]. This sequence operationalizes the diagnostic logic of the review, moving from inexpensive behavioral screening to definitive ambulatory confirmation and biomarker characterization, and finally to reassessment of nocturnal dipping as the measure of therapeutic success [12].

Detection at the level of the individual must be matched by action at the level of the population, because sleep and circadian health are public health concerns in their own right [81]. Structural measures hold particular promise. Aligning institutional schedules with adolescent circadian biology, most directly by delaying school start times, addresses the mismatch between intrinsic sleep timing and externally imposed routines that perpetuates sleep debt and circadian misalignment in this age group [7,82].

Prevention is also more effective when it is holistic, because the sedentary digital lifestyle harms more than the cardiovascular system. Beyond the cardiovascular system, a sedentary digital lifestyle also affects posture and musculoskeletal health [83], while regular physical activity is a protective counterweight associated with better wellbeing in adolescents [84]. Nighttime electronic-media use has itself been linked to sleep disturbance and depressive symptoms in adolescents, underscoring that the psychosocial and cardiovascular consequences of the digital exposome overlap [85]. A strategy aimed at digital chronotoxicity should therefore sit within a broader program that addresses sleep, physical activity, and psychosocial wellbeing together. The observational evidence linking screen exposure and disturbed sleep to adverse blood pressure and cardiometabolic phenotypes in young people is summarized in Table 5.

### 5.6. From Detection to Intervention

Because the purpose of detection is to enable action, the diagnostic pathway connects directly to a stratified, etiologically oriented management plan in which lifestyle and circadian correction precede pharmacology. A distinction should be drawn between interventions with good evidence for improving sleep and those specifically shown to restore nocturnal blood pressure dipping. Most measures below are supported primarily as sleep or circadian interventions; their capacity to convert a non-dipping into a dipping profile in adolescents rests largely on mechanistic reasoning and adult data and remains to be demonstrated in pediatric trials. In the context of chronodisruption, sleep hygiene functions as a precise therapeutic tool rather than generic advice [87]. Its central component is a digital curfew, the cessation of device use roughly 60 to 90 min before bedtime, complemented by relaxation techniques that lower sympathetic tone and by structured elements drawn from cognitive behavioral therapy for insomnia, an established behavioral approach for insomnia in young people [88]. Light filtering alone is insufficient, because it does not address the cognitive-arousal component of digital exposure.

Stabilizing the timing of sleep, including on weekends, is equally important, since it is the irregularity of sleep timing, and not only its duration, that perpetuates circadian misalignment. Regularizing the sleep schedule may help restore the dipper profile, an effect suggested mainly by adult and mechanistic data and not yet confirmed in pediatric trials, and one that appears at least partly independent of weight change. This is the essence of behavioral chronotherapy: the strategic use of external time cues to recalibrate the suprachiasmatic clock and, with it, the temporal architecture of the autonomic nervous system, thereby returning a cardiovascular system locked in nocturnal alertness to genuine hemodynamic rest [89].

When behavioral measures are insufficient, exogenous melatonin may be considered, under clinical supervision, as a sleep and circadian intervention rather than as an antihypertensive treatment. In adults, controlled-release melatonin has been associated with modest reductions in nocturnal blood pressure [90], mechanistically consistent with central inhibition of adrenergic outflow through MT1 receptors [19], stimulation of endothelial nitric oxide synthase through MT2 receptors [18], and antioxidant scavenging of reactive oxygen species [20]; its use specifically to lower blood pressure in adolescents is not supported by direct pediatric evidence and remains investigational. The principal pediatric caution concerns the interaction of melatonin with the hypothalamic–pituitary–gonadal axis, given its physiological inhibition of gonadotropin-releasing hormone; reassuringly, long-term use in children has not demonstrated significant delays in pubertal development, supporting a favorable safety profile under periodic monitoring [91].

In adults, antihypertensive therapy can be timed to target elevated nocturnal pressure, an approach of theoretical relevance here [89]; however, routine bedtime dosing of antihypertensive medication cannot be recommended in adolescents in the absence of pediatric outcome evidence, and any pharmacological decision remains individualized and specialist-led. Across all of these strategies, the appropriate measure of success is not the daytime office reading but the recovery of physiological nocturnal dipping on repeat ABPM, which makes ambulatory monitoring the natural follow-up standard as well as the diagnostic one [12,61]. The principal chronobiological interventions and their effects on the nocturnal blood pressure profile are summarized in Table 6.

### 5.7. Knowledge Gaps, Limitations, and Future Diagnostic Directions

Several gaps temper the conclusions that can currently be drawn. The first concerns causality. Most of the available evidence is observational, and direct studies relating adolescent screen exposure to the non-dipper ambulatory phenotype are scarce, so much of the chain remains inferential, connected through the intermediate steps of sleep disruption and metabolic change rather than demonstrated end to end [50,51]. A related limitation is confounding. Several conditions and exposures independently affect screen behavior, sleep, and nocturnal blood pressure and may account for part of the associations described here, including obesity, obstructive sleep apnea, chronic kidney disease, diabetes mellitus, secondary and monogenic hypertension, and medications or stimulants such as caffeine and energy drinks. Anxiety, physical inactivity, pubertal stage, and socioeconomic circumstances act as further shared determinants. Because much of the available evidence does not fully adjust for these factors, the independent contribution of nighttime digital exposure to the non-dipper phenotype cannot yet be isolated, and these variables should be measured and controlled for in future studies. A second gap concerns the nature of the exposure. Screen time is still frequently treated as a single quantity, whereas passive use and interactive use are likely to differ substantially in their autonomic impact, and future research should stratify cardiovascular risk by content type rather than by duration alone [31]. A third gap is quantitative: the melanopic threshold below which the adolescent circadian system is undisturbed has not been established, leaving exposure recommendations imprecise [77]. Finally, the reproducibility of a single ambulatory assessment is limited by night-to-night variability in sleep and behavior, which strengthens the case for continuous or repeated monitoring rather than reliance on an isolated study [69,92].

These gaps must be read alongside the limitations of the present review. As a narrative and integrative synthesis, it did not apply formal risk-of-bias scoring or quantitative pooling, and its reporting quality, while structured according to recognized criteria [15], reflects qualitative appraisal rather than meta-analysis. Some mechanistic links rest partly on adult or experimental data extrapolated to adolescents, pediatric ambulatory intervention trials remain few, and digital chronotoxicity is advanced here as a unifying construct that itself awaits formal operational definition and validation.

The corresponding research agenda follows directly. The construct of digital chronotoxicity should be standardized across its dimensions of intensity, timing, content, and cumulative exposure, so that it can be measured consistently. Cuffless and wearable estimates of blood pressure require validation against ambulatory monitoring in adolescents before they can support phenotype classification [57,69]. Prospective longitudinal cohorts and pediatric randomized trials of digital-hygiene and chronotherapeutic interventions should adopt the restoration of nocturnal dipping as a primary endpoint, rather than office blood pressure [88,89], and the predictive algorithms envisioned for digital biofeedback must be validated prospectively before clinical deployment [70]. Addressing these priorities would move the field from a plausible mechanistic narrative toward a validated, diagnostically actionable framework.

## 6. Conclusions

Arterial hypertension in adolescents can no longer be understood in isolation from the digital lifestyle that increasingly shapes it. This review has argued that intensive nighttime device use and the chronic sleep loss that accompanies it are not incidental behaviors but plausible contributors to chronodisruption, a state that can reorganize the circadian control of the cardiovascular system. We have framed this cumulative effect as digital chronotoxicity, a proposed integrative construct rather than an established clinical diagnosis, and traced a plausible course from an initial cellular trigger toward a measurable clinical endpoint.

The proposed pathway is internally coherent at each step. Evening blue light and cognitively arousing content can suppress melatonin and sustain sympathetic and neuroendocrine activation; the associated oxidative stress and eNOS uncoupling may impair nocturnal vasodilation; and volume retention plausibly compounds the effect. Sleep, which should be a period of hemodynamic recovery, is thereby converted into a window of active vascular risk. The proposed expression of this process is the loss of nocturnal blood pressure dipping, which, if unaddressed, may be accompanied by early structural changes such as left ventricular hypertrophy, arterial stiffening, and renal and cognitive involvement associated with premature cardiovascular aging.

The central message of this review is diagnostic. Because the decisive abnormality occurs during sleep, it is invisible to office measurement, and the evaluation of a hypertensive adolescent in the present era is therefore incomplete without both a deliberate digital and sleep history and 24 h ambulatory blood pressure monitoring as the reference standard. Around that standard, a layered panel of cardiac, vascular, renal, autonomic, and neurocognitive biomarkers and an emerging generation of wearable and artificial-intelligence tools offer the means to detect the at-risk phenotype early and, increasingly, to anticipate it.

Management should evolve accordingly, from generic salt restriction toward behavioral and pharmacological chronotherapy whose success is judged by the recovery of dipping rather than by daytime values. Reframed in this way, pediatric hypertension may be viewed as a chronobiological cardiovascular process that can begin early and may therefore be detectable, and potentially preventable, early. Realizing this prevention will require treating the nocturnal circadian phenotype, and the digital behavior that may disturb it, as central rather than peripheral to adolescent cardiovascular health. At the same time, the causal, screening, and treatment propositions advanced here remain provisional: they require confirmation by direct prospective pediatric studies that combine objective measurement of screen exposure, formal sleep assessment, and ambulatory blood pressure monitoring before they can support firm clinical recommendations.

## Figures and Tables

**Table 1:** Chronobiological mechanisms of digital chronotoxicity, their cardiovascular consequences, and the markers that detect them.

Digital Exposure/Trigger	Chronobiological Mechanism	Cardiovascular Effect	Detectable Marker/Tool	Refs.
Evening blue light (460–480 nm)	ipRGC → SCN → pineal: melatonin suppression; loss of MT2 vasodilation and antioxidant protection	↑ nocturnal vascular tone, ↓ NO bioavailability	Salivary/plasma melatonin; melanopic light exposure	[18,19,20,24,25]
Interactive/competitive content (gaming, social media)	Dopaminergic reward → shift toward sympathetic predominance; failure of vagal withdrawal	↑ heart rate and cardiac output; daytime-level nighttime BP	HRV (LF/HF ratio)	[30,35,36]
Chronic sleep deprivation	HPA activation → elevated evening cortisol (mineralocorticoid effect)	Na^+^/H_2_O retention, ↑ intravascular volume	Evening/late-night cortisol	[42]
Sleep loss with low-grade inflammation	↑ ROS (superoxide) → peroxynitrite → eNOS uncoupling	Endothelial dysfunction; arterial remodeling and stiffening	hsCRP, IL-6; endothelial indices; PWV, cIMT	[44,45,46]
Sleep restriction (metabolic)	↓ insulin sensitivity → hyperinsulinemia (sympathetic + tubular Na reabsorption); ↓ leptin/↑ ghrelin	Salt-sensitive volume expansion	Fasting insulin/HOMA-IR; cardiometabolic profile	[42,50,51]
Convergent outcome	Loss of sympathetic withdrawal + volume expansion + stiffening	Loss of nocturnal dipping → non-dipper phenotype; early vascular aging	24 h ABPM (dipping %); PWV, cIMT, LV mass index, microalbuminuria	[11,12]

**Table 2:** Circadian blood pressure phenotypes: ambulatory definitions, prognostic significance, and relevance to digital chronotoxicity.

Phenotype	Nocturnal Dipping (ABPM Criterion)	Prognostic Significance	Relevance to Digital Chronotoxicity	Refs.
Dipper	Nocturnal fall ≥ 10% (and ≤20%)	Physiological, cardioprotective; reference pattern	Preserved when the circadian rhythm is intact	[52]
Non-dipper	Nocturnal fall < 10%	Sustained nocturnal load; predicts organ damage and adult hypertension	Core phenotype hypothesized to result from digital chronotoxicity; nocturnal load predicts organ damage and adult hypertension	[12,52,54]
Nocturnal hypertension	Asleep BP above age/height threshold (may occur with or without reduced dipping)	Elevated absolute nocturnal load; predicts organ damage	Plausible consequence of sustained nighttime exposure	[12,14]
Masked hypertension	Normal office BP with elevated out-of-office (ambulatory/home) BP	Often undetected; associated with organ damage	May coexist with, but is distinct from, non-dipping	[14,53]
Extreme dipper	Nocturnal fall > 20%	Linked to nocturnal hypoperfusion and cerebrovascular risk	Less directly related; included for completeness	[52]
Reverse dipper/riser	Night BP exceeds day BP (negative dipping)	Highest cardiovascular risk of all patterns	Plausibly more frequent in adolescents with disordered sleep and heavy nighttime screen use (hypothesis; not established by direct data)	[56]

**Table 3:** Comparison of blood pressure measurement modalities for detecting the non-dipper phenotype and masked hypertension in adolescents.

Modality	What It Captures	Detects Non-Dipping	Detects Masked HTN	Key Strengths	Key Limitations	Refs.
Office BP	A few readings during waking clinic hours	No	No	Simple, universal, low cost	Misses nocturnal phenotype and masked HTN; white-coat effect; poor reproducibility	[53]
Home BP monitoring (HBPM)	Repeated self-measured awake readings at home	No	Partial	Accessible, repeatable, reduces white-coat effect	Cannot capture the asleep period or dipping; technique-dependent	[13]
24 h ABPM	Automated awake and asleep readings over 24 h	Yes (reference)	Yes	Only method capturing asleep BP and dipping; derived indices; correlates with organ damage	Tolerability in children; cuff-induced sleep disturbance; imperfect night-to-night reproducibility; cost	[12,13,53,57,58,61]
Wearables (cuffless PPG, HRV, actigraphy)	Continuous autonomic and sleep signals; estimated BP	Emerging/ indirect	Potential	Continuous, real-time, scalable; good adherence; captures HRV	BP estimation not yet validated to ABPM standard; accuracy/regulatory gaps	[36,57]

**Table 4:** Biomarkers of early hypertension-mediated organ damage associated with an elevated nocturnal blood pressure load in adolescents.

Domain	Biomarker	Modality	Abnormal Finding	Reflects/Predicts	Refs.
Cardiac	Left ventricular mass index	Echocardiography	Increased indexed LV mass; tracks nocturnal more than daytime BP	LVH; predictor of MI/stroke in young adults	[62,63]
Cardiac	Diastolic function	Tissue Doppler imaging	Impaired relaxation before overt hypertrophy	Preclinical cardiac involvement	[63]
Vascular	Pulse wave velocity	Tonometry/oscillometry	Elevated vs. age norms	Arterial stiffness; accelerated vascular aging	[63,64]
Vascular	Carotid intima–media thickness	Carotid ultrasound	Increased thickness	Subclinical arteriopathy	[63]
Vascular	AASI; average real variability	Derived from 24 h ABPM	Elevated indices	Arterial stiffness and BP lability; inflammation-linked	[58,65]
Renal	Microalbuminuria	Spot urinary albumin	Elevated urinary albumin	Glomerular hyperfiltration; systemic endothelial dysfunction	[22,63]
Autonomic	Heart rate variability (LF/HF)	ECG/wearable	Increased LF/HF, reduced vagal tone	Non-specific autonomic marker; supportive, not diagnostic of non-dipping	[36]
Neurocognitive	Attention/executive function	Neuropsychological testing	Lower test performance	CNS impact of nocturnal hypertension	[67,68]

**Table 5:** Representative studies linking screen exposure and sleep disturbance to blood pressure and cardiometabolic phenotypes in children and adolescents.

Study	Design	Population	Exposure	Outcome	Main Finding	Ref.
Jahangiry et al.	Systematic review and dose–response meta-analysis	Children and adolescents	Screen time	Metabolic syndrome (incl. elevated BP)	Higher screen time associated with greater metabolic syndrome risk, dose-dependently	[3]
Lange et al.	Longitudinal (pandemic period)	Children and adolescents	Screen-saturated pandemic environment	BMI and systolic BP	Parallel increases in BMI and systolic BP during the pandemic	[5]
St-Onge et al.	Scientific statement (AHA)	Adolescents	Sleep duration and quality	Cardiometabolic markers	Shorter, poorer sleep linked to an adverse cardiometabolic profile	[48]
Li et al.	Longitudinal cohort	Adolescents	Screen time and sleep	Depressive symptoms	Sleep partly mediates the association between screen time and depressive symptoms	[50]
Pompeia et al.	Observational	Early adolescents	Social jetlag	Cardiometabolic latent traits	Greater social jetlag linked to adverse cardiometabolic traits	[76]
Jansen et al.	Prospective cohort	Adolescents	Bedtime timing	Blood pressure	Later bedtimes prospectively associated with higher blood pressure	[86]
Horner et al.	Cohort	Children and adolescents	Screen time	Cardiometabolic/CV risk	Greater screen time associated with higher cardiometabolic and cardiovascular disease risk	[6]
King et al.	Experimental	Adolescents	Prolonged violent video-gaming	Sleep	Evening gaming delayed sleep onset and reduced sleep, supporting an arousal (not merely photic) pathway	[32]

**Table 6:** Chronobiological and behavioral interventions for the non-dipper phenotype and their effect on nocturnal blood pressure.

Intervention	Mechanism/Rationale	Effect on Nocturnal BP and Dipping	Refs.
Digital curfew (devices off 60–90 min before sleep)	Reduces evening light and arousal; preserves melatonin, lowers sympathetic tone	May support the nocturnal melatonin rise; effect on dipping not established in adolescents	[87,88]
CBT-I and relaxation techniques	Target cognitive arousal and insomnia	Improve sleep continuity; reduce pre-sleep sympathetic activation	[88]
Regular sleep schedule (including weekends)	Reduces social jetlag and circadian misalignment	May support dipping recovery (adult/mechanistic evidence; not confirmed in adolescents)	[76,89]
Blue-light filtering (lenses/night modes)	Reduces melanopic exposure	Partial melatonin preservation; insufficient alone	[78,80]
Controlled-release exogenous melatonin	Chronobiotic; MT1 sympatholytic, MT2 eNOS/NO, antioxidant	Reduces nocturnal BP in adults; pediatric antihypertensive effect not established	[18,19,20,90,91]
Chronopharmacology (timed antihypertensive)	Aligns drug action with the nocturnal pressure peak	Adult evidence; not established in adolescents	[12,61,89]
ABPM-guided follow-up	Objective endpoint	Restoration of dipping proposed as an objective endpoint	[12,53,61]

## Data Availability

No new data were created or analyzed in this study. Data sharing is not applicable to this article.

## Abbreviations

The following abbreviations are used in this manuscript:

AASI	ambulatory arterial stiffness index
ABPM	ambulatory blood pressure monitoring
AI	artificial intelligence
BMI	body mass index
BP	blood pressure
CBT-I	cognitive behavioral therapy for insomnia
cIMT	carotid intima–media thickness
CNS	central nervous system
ECG	electrocardiography
eNOS	endothelial nitric oxide synthase
HBPM	home blood pressure monitoring
HMOD	hypertension-mediated organ damage
HOMA-IR	homeostatic model assessment of insulin resistance
HPA	hypothalamic–pituitary–adrenal (axis)
HRV	heart rate variability
hsCRP	high-sensitivity C-reactive protein
IL-6	interleukin-6
ipRGC	intrinsically photosensitive retinal ganglion cell
LF/HF	low-frequency/high-frequency ratio
LV	left ventricular
LVH	left ventricular hypertrophy
MI	myocardial infarction
MT1	melatonin receptor type 1
MT2	melatonin receptor type 2
NO	nitric oxide
PPG	photoplethysmography
PWV	pulse wave velocity
ROS	reactive oxygen species
SANRA	Scale for the Assessment of Narrative Review Articles
SCN	suprachiasmatic nucleus
